# Association between tissue stress reaction and ACE2/TMPRSS2 expression in endometria of reproductive aged women before and during Covid-19 pandemic

**DOI:** 10.1186/s12905-023-02378-0

**Published:** 2023-05-04

**Authors:** Kanae Ogawa, Khaleque N. Khan, Akemi Koshiba, Akira Fujishita, Go Horiguchi, Satoshi Teramukai, Kyoko Itoh, Sun-Wei Guo, Taisuke Mori

**Affiliations:** 1 Department of Obstetrics and Gynecology, Graduate School of Medical Science, Kyoto Prefectural University of Medicine, 465 Kajii-Cho, Kamigyo-Ku, 602-8566 Kyoto, Japan; 2 The Clinical and Translational Research Center, Graduate School of Medical Science, Kyoto Prefectural University of Medicine, 465 Kajii-Cho, Kamigyo-Ku, 602-8566 Kyoto, Japan; 3 Department of Gynecology, Saiseikai Nagasaki Hospital, Nagasaki, Japan; 4 Department of Biostatistics, Graduate School of Medical Science, Kyoto Prefectural University of Medicine, Kyoto, Japan; 5 Department of Pathology and Applied Neurobiology, Graduate School of Medical Science, Kyoto Prefectural University of Medicine, Kyoto, Japan; 6 Shanghai Obstetrics and Gynecology Hospital, Fudan University, Shanghai, China

**Keywords:** Covid-19 pandemic, Endometrium, ACE2/TMPRSS2, NK1R/ADRB2, Anxiety/stress, Pre-pandemic, In-pandemic

## Abstract

**Background:**

A potential concern has been raised regarding fertility and reproductive outcome during the Covid-19 pandemic with growing stress and anxiety. However, information on the association between tissue stress reaction and expression profiles of SARS-CoV-2 viral entry proteins, ACE2 and TMPRSS2, in endometria collected from women before (pre-pandemic) and during the Covid-19 pandemic (in-pandemic) is unknown. We aim to investigate the relationship between the expression of stress-reactive proteins and of ACE2 and TMPRSS2 in endometria collected from women during these two different time frames.

**Methods:**

We retrospectively retrieved tissue blocks of endometrial samples from 25 women in 2019 (pre-pandemic) and 25 women in 2020 (in-pandemic) who underwent hysterectomy for different gynecological indications. Immunohistochemical analysis was performed with endometrial tissue samples that were collected before and during the pandemic, using respective antibodies targeting ACE2/TMPRSS2, ADRB2 and NK1R (stress and anxiety receptor markers, respectively). The quantification of immunoreactive cells for each marker was calculated by the immunoreactive score (IRS) analysis. This retrospective cohort study was limited to small sample size.

**Results:**

No significant differences in the IRS of ACE2 and TMPRSS2 were found between the endometria that were collected before and during the pandemic with a lack of correlation between ACE2 and TMPRSS2 expression in respective endometria (*r* = 0.11, pre-pandemic; *r* = 0.04, in-pandemic). The immunostaining levels of stress marker, ADRB2 were significantly higher in the endometria of in-pandemic group (*p* = 0.015) comparing to that of pre-pandemic group. Pearson’s correlation coefficient analysis showed a significant correlation in the expression between ADRB2 and TMPRSS2 (*r* = 0.41, *p* = 0.042) in the endometria of in-pandemic group but not in the pre-pandemic group.

**Conclusion:**

The rise in stress and anxiety among women during current pandemic may elicit substantial amount of tissue stress reaction with consequent increase in the expression of SARS-CoV-2 viral entry proteins in their endometria. A lack of correlation between ACE2 and TMPRSS2 expression in endometria may reassure women in their reproductive age that they are not more susceptible to infection by SARS-CoV-2 and suggest that stressful women during this pandemic can safely decide to conceive naturally or by artificial reproductive technology.

**Supplementary Information:**

The online version contains supplementary material available at 10.1186/s12905-023-02378-0.

## Introduction

Coronavirus disease 19 (Covid-19), caused by severe acute respiratory syndrome coronavirus 2 (SARS-CoV-2), has been regarded as the largest and deadliest pandemic since the 1918 influenza pandemic [1]. With the first case with Covid-19 disease reported in Wuhan, China, in December 2019 [1], Japan confirmed its first case on January 15, 2020 [2]. As the Covid-19 pandemic quickly swept the entire globe claiming millions of lives, people around the world watched the pandemic unfolding in horror, disbelief, and fear and experience much stress and anxiety. This pandemic also has wreaked havoc in its way, causing disruption and chaos in economy, education, healthcare, let alone social life. Among the vulnerable population, many women in their reproductive age may be concerned of their fertility and reproductive outcome during the Covid-19 pandemic with growing stress, frustration and anxiety. In addition, pregnant women and their fetuses have traditionally represented as a high-risk population during viral pandemics [3].

Several recent studies demonstrated an association between Covid-19 pandemic and abnormal mental health. The study from Turkey showed that the Covid-19 pandemic caused depression, anxiety, and serious sleep disorders in pregnant women [4]. According to a study conducted in Brazil, anxiety and depression were the most prevalent psychiatric symptoms of Covid-19 in the general population [5]. In addition, a positive correlation was found between depression, anxiety, and perceived stress among hospitalized patients with Covid-19 [6–8]. Most recently, the percentage of US adults who reported feelings of anxiety/stress and depression peaked to 41% (July 2020) and 42.6% (November 2020) and remained high through June 2022. This rate was more than four times than in 2019 [9–11]. Despite all these published reports, our knowledge is insufficient on the association between exogenous mental stress/anxiety and endogenous stress reaction in human endometrium. Our current study may clarify, at least in part, this unclear issue. Here, we propose a hypothesis whether suffering from a variable exogenous stress/anxiety in response to Covid-19 pandemic might be involved in tissue stress reaction in the endometria and its link with the possible change in the expression patterns of SARS-CoV-2 cell entry receptors in the endometria of women in their reproductive age.

SARS-CoV-2 is a single-stranded positive sense RNA virus and infected individuals can be either asymptomatic or present mild to severe symptoms. SARS-CoV-2 binds to angiotensin-converting enzyme 2 (ACE2), a key viral entry receptor on the host cell through spike (S) glycoprotein located on the surface of the virus [12, 13]. The entry of the virus into the host cells is also mediated by host proteases such as transmembrane serine protease 2 (TMPRSS2) in addition to ACE2 [12, 13]. TMPRSS2 is needed to cleave the viral S protein to induce a conformational change to S that allows for permanent fusion of viral and host cell membrane and permits efficient entry into the host cell [14, 15]. The importance of TMPRSS2 has been confirmed by the fact that its inhibition blocks SARS-CoV-2 entry and spread in infected cells [13, 14]. Although cell-specific expression of ACE2 and TMPRSS2 receptors in the female reproductive organs have been demonstrated [16], information on ACE2 and TMPRSS2 protein expression in human endometria collected from two different time frames (pre-pandemic and during pandemic) is lacking. Our proposed study on the protein expression of these two SARS-CoV-2 viral entry proteins in endometria may illuminate our knowledge.

One line of evidence indicated that a variable amount of physical or emotional stress results in the secretion of various bioactive molecules such as catecholamines and substance-P from the adrenal glands and brain cells, respectively [17–20]. Catecholamines are known to suppress cell-mediated immunity and promotes angiogenesis, cell proliferation, metastasis in animal models [18–20]. We speculate that if an increased tissue stress reaction enhances cellular entry of SARS-CoV-2 by up-regulating expression of ACE2 and TMPRSS2 receptors in endometria, it may further impact on successful implantation and placentation. In addition, a state of physical or emotional stress in women during this pandemic may exacerbate the growth and progression of any coexistent disease [18, 19]. Some recent studies indicated that suffering from mental anxiety and stress during Covid-19 pandemic may result in variable menstruation-related problems such as menstrual irregularity, short menstrual cycle and/or decreased amount of menstrual blood loss [21–23].

A parallel investigation in the expression of stress/anxiety receptors and their association with SARS-CoV-2 entry proteins in endometria of pre-pandemic and in-pandemic (during the pandemic) women is necessary to clarify this unclear issue. After extensive literature search, we did not find any information on the expression pattern of ACE2 and TMPRSS2 protein in endometria of women collected during Covid-19 pandemic (in-pandemic) comparing them to that in the endometria of women collected during the pre-pandemic period. We can learn further information on this issue from our current study. It has been reported that SARS-CoV-2-induced altered expression of ACE2 in brain cells may be associated with depression and anxiety by decreasing levels of serotonin and dopamine [19, 20]. We speculate that a constant tissue stress reaction in the endometria, in response to physical and/or psychological, stress/anxiety/fear, of women during Covid-19 pandemic may change the expression profiles of ACE2 and TMPRSS2, stress/anxiety receptors or at least may aggravate endometrial inflammatory reaction by increasing the accumulation of innate immune cells. In addition, possible increased tissue expressions of catecholamine receptors in the endometria during the Covid-19 pandemic may provide clues to understand the mechanisms underlying pandemic-related menstrual disturbances as reported recently [21–23].

Based on the lack of enough information and limitation of previous studies three research questions may form the core of our proposed research plan: (1) Would proper retrieval of endometrial tissue samples from women of two different time frames (pre-pandemic and in-pandemic) and analysis of a panel of markers in respective endometria have some potential in reproductive aged women? (2) Would analysis of stress-reactive proteins and SARS-CoV-2 viral entry proteins in endometria address an association between them? (3) Would analysis of different immunocompetent cells in pre-pandemic and in-pandemic endometria address a mechanistic link to clarify enhanced tissue stress reaction in endometria collected during Covid-19 pandemic?

In an attempt to address these issues, we retrospectively retrieved tissue blocks of endometrial samples that were collected from two groups of women who underwent hysterectomy during the Covid-19 pre-pandemic period (2019, pre-pandemic group) and during the period of pandemic (2020, in-pandemic group). We used these two sets of endometrial samples to examine the following issues by immunohistochemical analysis: (1) any change in the expression pattern of SARS-CoV-2 receptors, ACE2 and TMPRSS2, (2) any change in the expression pattern of receptors related to stress and anxiety such as adrenergic receptor β2 (ADRB2), one of the widely reported receptors for catecholamines and neurokinin 1 receptor (NK1R), a recognized functional receptor for substance-P, respectively, (3) any change in the accumulation of innate immune cells in the endometria such as CD68-stained macrophages (Mφ) and myeloperoxidase (MPO)-stained neutrophils. We selected these two innate immune cells in our study because they act as first line defensive cells in response to any emergency crisis in external or internal environment of human body, and (4) age-dependent distribution in the expression profiles of ACE2 and TMPRSS2, ADRB2 and NK1R, and CD68 and MPO in the endometria that were collected before and during Covid-19 pandemic.

## Materials and methods

### Patients

This was a retrospective observational cohort study with endometrial samples that were collected from two groups of women who underwent hysterectomy in the Covid-19 pre-pandemic period (2019, pre-pandemic group) and during the period of Covid-19 pandemic (2020, in-pandemic group).

### Collection of endometrial samples

Paraffin-embedded tissue blocks of human endometrium were retrieved from 50 patients undergoing hysterectomy for different gynecological diseases. All surgeries were performed at the department of Obstetrics and Gynecology, Kyoto Prefectural University of Medicine. Among 50 cases, 25 cases underwent surgery between January 2019 and December 2019 (pre-pandemic) and 25 cases underwent surgery between February 2020 and December 2020 (in-pandemic). The baseline demographic profiles of these two groups of women such as age, body mass index (BMI), gravidity, parity, menstrual cycle, hormonal medication, past or current smoking, coexistent diseases, surgical interventions, and pathological diagnosis were collected from medical records. BMI is the weight in kilogram divided by the square of the height in meters. Menstrual cycle was determined by the last menstrual period or pathological findings. All endometrial specimens were collected in accordance with the guidelines of the Declaration of Helsinki and were approved by the Institutional Review Board of Kyoto Prefectural University of Medicine (IRB No. ERB-C-1445–3). A written informed consent was not necessary due to retrospective nature of the study.

### Antibodies used

We performed immunohistochemical analysis using respective antibodies against target antigens in the serial section of endometrial samples as follows: SARS-CoV-2 cell entry proteins, ACE2 (MAB-933), mouse monoclonal, 1:200; TMPRSS2 (ab214462), rabbit polyclonal, 1:100; antibody for receptors against substance-P, NK1R (NB300-119), rabbit polyclonal, 1:200; antibody for catecholamines, ADRB2 (ab137494), rabbit polyclonal, 1:200; CD68, (Mφmarker, M0814), mouse monoclonal, 1:200; myeloperoxidase (MPO, marker of neutrophils, ab9535), rabbit polyclonal, 1:50 dilution. A complete list of primary antibodies, concentrations used for each antibody, clonality, manufacturing companies, and respective positive controls are shown in Table 1.

**Table 1:** List of antibodies used in current study

Name of antibody	Catalog number	Clonality	Host	Conc. used	Name of Company	Positive control
ACE2	MAB-933	monoclonal	mouse	1:200	R&D systems	testis, kidney
TMPRSS2	ab214462	polyclonal	rabbit	1:100	abcam	testis, kidney
NK1R	NB300-119	polyclonal	rabbit	1:200	Novus biologicals	human brain^a^
ADRB2	ab137494	polyclonal	rabbit	1:200	abcam	melanoma
CD68	M0814	monoclonal	mouse	1:200	Dako	lymph node
MPO	ab9535	polyclonal	rabbit	1:50	abcam	lymph node

*ACE2* angiotensin-converting enzyme 2, *TMPRSS2* trans-membrane serine protease 2, *NK1R* neurokinin receptor 1, *ADRB2* beta 2 adrenergic receptor (C-terminal), *CD68* marker of macrophages, *MPO* myeloperoxidase, marker of neutrophils

^a^Basal nucleus or Meynert neurons of human brain tissue

#### Immunohistochemistry

The details of immunohistochemical staining procedures are described elsewhere [24, 25]. Briefly, 4 μm thick paraffin-embedded tissue sections were deparaffinized in xylene and rehydrated in graded alcohols and distilled water. Antigen retrieval was done for respective antigens. After immersion in 0.3% H_2_O_2_-methanol to block endogenous peroxidase activity (30 min), sections were pre-incubated with blocking buffer for 1 h and then incubated overnight at 4℃ with respective primary antibodies. Sections then were incubated with the secondary anti-rabbit or anti-mouse antibody (90 min, room temperature) followed by visualization with diaminobenzidine-H_2_O_2_. Finally, the tissue sections were counterstained with Mayer’s hematoxyline, dehydrated with serial alcohols, cleared in xylene, and mounted. A parallel staining of negative control for each slide was prepared and was incubated without primary antibody.

#### Quantification of immunoreactive cells

The immunoreactive CD68-, and MPO- stained cells were counted in five different fields of one section by light microscopy at moderate magnification (× 200) and was expressed as the mean Mφ and MPO number per high power field (HPF) in one specimen. We represented Mφ- and MPO-stained cells in stromal compartment. The immunoreactivity for each of ACE2, TMPRSS2, ADBR2 and NK1R in samples of endometria was quantified by immunoreactive score (IRS) system as reported elsewhere [26, 27]. IRS is calculated by multiplying the staining intensity (category A) and the percentage of immunoreactive cells (category B). The staining intensity was graded as 0 (no staining), 1 (weak immunostaining), 2 (moderate immunostaining), and 3 (strong immunostaining). The percentage of immunoreactive cells was graded as 0 (0%), 1 (< 10%), 2 (10 ~ 50%), 3 (50 ~ 80%), 4 (> 80%). Multiplication of category A and B resulted in an IRS ranging from 0–12. We represented IRS in each endometrial sample by combined immunoreactive cells in surface epithelium, glandular epithelium and stromal compartment. We calculated the mean IRS of five different fields of one section by light microscopy at moderate magnification (× 200). Counting of CD68- and MPO-stained cells and measurement of IRS in endometrial samples were performed by a single investigator (KO) who was blind to the clinical data.

### Statistical analysis

All results are expressed as mean ± SD, mean ± SEM or median and interquartile ranges. The clinical characteristics of the subjects between groups were analyzed by one-way analysis of variance (ANOVA). Any difference in the expression of ACE2, TMPRSS2, NK1R, ADRB2 and number of CD68- or MPO-stained cells between groups was analyzed by the Mann–Whitney U test. Continuous variables were compared between groups using Wilcoxon rank sum test and categorical variables were compared using Fisher’s exact test. Kruskal–Wallis test was used to determine any difference among groups. Any correlation in the expression of different markers between groups was analyzed by Pearson product-moment correlation coefficient. Analysis of covariance (ANCOVA) with different confounding variables (age, BMI, gravidity, parity, smoking, hormonal therapy, and menstrual cycle) as covariates was used to compare different markers between groups. The distribution of each marker between groups was expressed using the box and whisker plots with the medians and inter-quartile range (IQR). A value of *p* < 0.05 was considered statistically significant. Data analysis was conducted using SAS software version 9.4 (SAS Institute Inc. Cary, NC, USA).

## Results

The clinical characteristics of patients before the Covid-19 pandemic (pre-pandemic) and during the Covid-19 pandemic (in-pandemic) from whom endometrial samples were collected for the analysis by immunohistochemistry are shown in Table 2. Continuous variables and categorical variables between groups indicated that there were no significant differences in the distribution of age, BMI, gravity, parity, phases of menstrual cycle, history of hormonal medication and smoking between these two groups of women (Table 2). The details of coexisting diseases, surgical procedures and indications of surgery for these cases are shown in Table 2 and displayed no remarkable difference in their distribution between groups. The pathological diagnosis, as obtained from medical records, of all cases from whom endometria were collected before and during Covid-19 pandemic are shown in Suppl. Table 1.

**Table 2:** Clinical profiles of cases from whom endometrial samples were collected before and during Covid-19 pandemic for immunohistochemical study

	Pre-pandemic (*n* = 25)	In-pandemic (*n* = 25)	*P* value
Age in years (mean ± SD)	44.1 ± 2.68	42.2 ± 4.42	0.068
Median (range, years)	44 (39–51)	43 (32–53)	
BMI (kg/m^2^)) (mean ± SD)	23.7 ± 5.27	23.3 ± 5.80	0.528
Median (range)	21.9 (15.7–41.9)	22 (18.3–46.5)	
Gravity (No.) (mean ± SD)	1.6 ± 1.29	2.1 ± 1.9	0.356
Median (range, No.)	2 (0–5)	2 (0–7)	
Parity (No.) (mean ± SD)	1.3 ± 1.06	1.4 ± 1.23	0.792
Median (range, No.)	1 (0–4)	1 (0–5)	
Menstrual cycle:			
P/O/S/M/A/unclear	2/2/16/1/2/2	1/1/14/0/2/7	0.519
Hormonal therapy: yes/no/unknown	7/18/0	12/13/0	0.244
Smoking: yes/no	11/14	7/18	0.377
Coexisting diseases:			
uterine myoma: yes/no	24/1	19/6	0.098
endometriosis: yes/no	4/21	3/22	1.000
adenomyosis: yes/no	1/24	4/21	0.349
none: yes/no	1/24	4/21	0.349
*Surgical procedures:			
TLH: yes/no	19/6	19/6	1.000
BTR: yes/no	14/11	14/11	1.000
RASH: yes/no	6/19	6/19	1.000
LSCP: yes/no	1/24	0/25	1.000
*Indication of surgery:			
CIN2-3/myoma/MBT/PU/	6/18/2/1/1/4	5/15/0/0/0/1/7	-
SCC/AIS/hypermenorrhea			

Continuous variables were compared between groups using Wilcoxon rank sum test and categorical variables were compared using Fisher’s exact test

P, proliferative phase, O, ovulatory phase, S, secretory phase, M, menstrual phase, A, amenorrhea; TLH, total laparoscopic hysterectomy, BTR, bilateral tubal resection, RASH, robot-assisted simple hysterectomy, LSCP, Laparoscopic sarcocolpopexy; CIN2-3, cervical intraepithelial neoplasia, grade 2 or 3, MBT, mucinous or seromucinous borderline tumor, PU, prolapse of the uterus, SCC, invasive squamous cell carcinoma, AIS, cervical adenocarcinoma in situ; *indicates cases coexistent with other surgical procedure or indication of surgery

### Immunoreactivity of ACE2 and TMPRSS2 in endometria

**Fig. 1:**
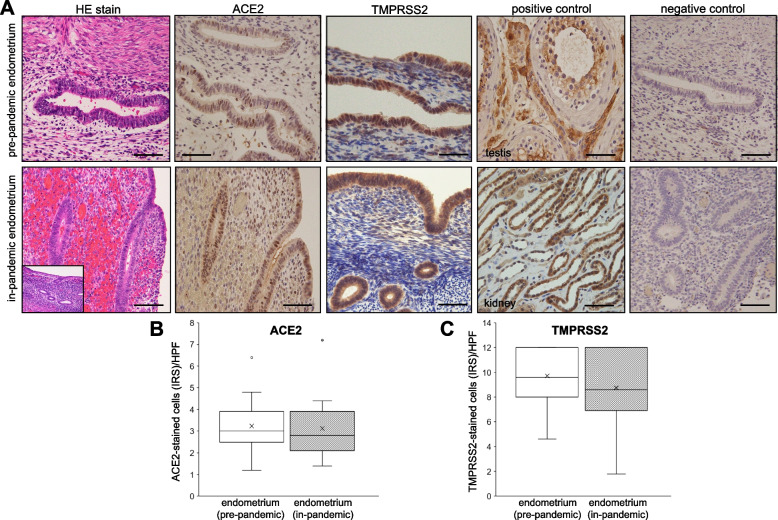
**A** An image of slides showing the hematoxyline eosin (HE)-stained and immunohistochemical analysis of angiotensin converting enzyme 2 (ACE2)- and trans-membrane serine protease 2 (TMPRSS2)-stained cells in the pre-pandemic (upper row) and in-pandemic endometria (lower row) with corresponding positive controls (testis for ACE2 and kidney for TMPRSS2) and negative controls. **B** and **C** Shows the immunoreactivity of ACE2 and TMPRSS2 as measured by the immunoreactive score (IRS) in pre-pandemic and in-pandemic endometria. The procedure of IRS measurement is described in methods. Mann–Whitney U test indicated no significance difference in the expression of ACE2 and TMPRSS2 between these two groups of endometria. The boxes represent the interquartile ranges and horizontal lines in the boxes represent median values. Scale bar = 50 μm for each slide

We analyzed the result of ACE2 and TMPRSS2 expression in respective endometrium (pre-pandemic and in-pandemic) derived from women who underwent hysterectomy. We found weak to moderate expression of ACE2 in surface epithelium, glands and stromal compartments. In contrast, TMPRSS2 showed moderate to strong immunoreactivity to surface epithelium, gland cells and stromal cells of each group of endometria (Fig. 1A). A parallel positive control from testis and kidney and negative controls were used for each of ACE2 and TMPRSS2, respectively (Fig. 1A). Mann–Whitney U test indicated that there were no significant differences in the immunoreactive scores (IRS) of ACE2 and TMPRSS2 between pre-pandemic endometria and in-pandemic endometria (Fig. 1B and C). Pearson correlation coefficient analysis confirmed that there was no significant correlation between expression of ACE2 and TMPRSS2 in endometria that were collected in 2019 (*r* = 0.11) and 2020 (*r* = 0.04) (Table 3).

**Table 3:** Correlation between expression of ACE2 and TMPRSS2 in endometria that were retrieved before (pre-pandemic) and during (in-pandemic) Covid-19 pandemic

	N	*r* value^a^	*P* value
Pre-pandemic (2019)	25	0.11	0.587
In-pandemic (2020)	25	0.04	0.838

*ACE2* angiotensin-converting enzyme 2, *TMPRSS2* trans-membrane protease serine protease 2

^a^Data were analyzed by Pearson product-moment correlation coefficient

There was no difference in the expression pattern of ACE2 and TMPRSS2 in either endometria across the phases of the menstrual cycle (data not shown). In addition, there was no difference in protein expression patterns of ACE2 and TMPRSS2 across the different tissue compartments (endometrium vs. myometrium) (data not shown). In fact, to our knowledge, there is no study until now that compared protein expression of SARS-CoV-2 cell entry receptors between different compartments of uterus.

### Immunoreactivity of NK1R and ADRB2 in endometria

**Fig. 2:**
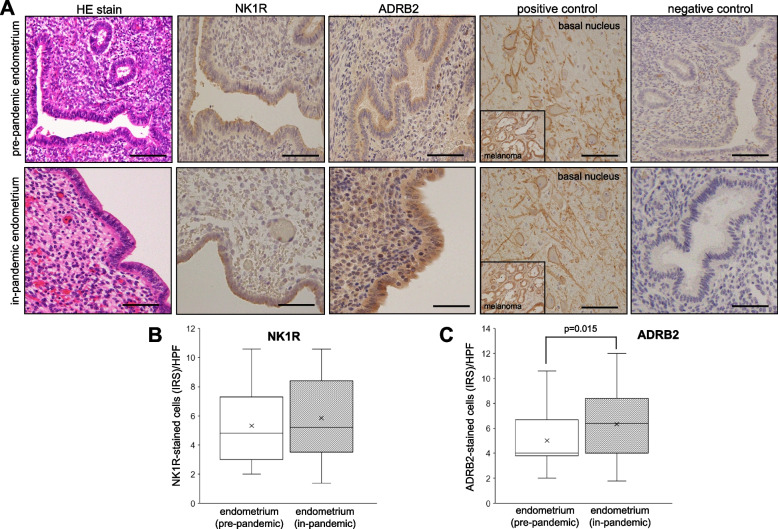
**A** An image of slides showing the hematoxyline eosin (HE)-stained and immunohistochemical analysis of neurokinin receptor 1 (NK1R)- and β2-adrenergic receptor (ADRB2)-stained cells in the pre-pandemic (upper row) and in-pandemic endometria (lower row) with corresponding positive controls (basal nucleus, Meynert neurons of human brain tissue for NK1R and melanoma for ADRB2) and negative controls. The immunoreactivities of NK1R **B** and ADRB2 **C** are shown as measured by the immunoreactive score (IRS) in pre-pandemic and in-pandemic endometria. The procedure of IRS measurement is described in methods. Mann–Whitney U test indicated that while no significance difference in the endometrial expression of NK1R was observed between the groups, ADRB2 expression in the endometria was significantly higher in the in-pandemic group than that in pre-pandemic group (*p* = 0.015) (**C**). The boxes represent the interquartile ranges and horizontal lines in the boxes represent median values. Scale bar = 50 μm for each slide

We were curious to know the expression patterns of anxiety/stress related receptors in endometria that were collected from women in 2019 (pre-pandemic) and 2020 (in-pandemic). As for NK1R, a weak to moderate immunoexpression was found in the surface epithelium, gland cells and stromal cells in respective endometria. In contrast, a moderate to strong immunoreaction of ADRB2 was observed in the surface epithelium, glands cells and stromal cells (Fig. 2A). A parallel positive control from basal nucleus (Meynert neurons of human brain tissue) for NK1R and melanoma tissue for ADRB2 and negative controls were used (Fig. 2A). Mann–Whitney U test revealed that while immunoreactivities of NK1R as measured by IRS were not significantly different between groups (Fig. 2B), the IRS of ADRB2 were significantly higher in the endometria of in-pandemic group comparing to that of pre-pandemic group (*p* = 0.015) (Fig. 2C).

### Correlation between NK1R or ADRB2 and ACE2 or TMPRSS2 expressions in endometria

**Fig. 3:**
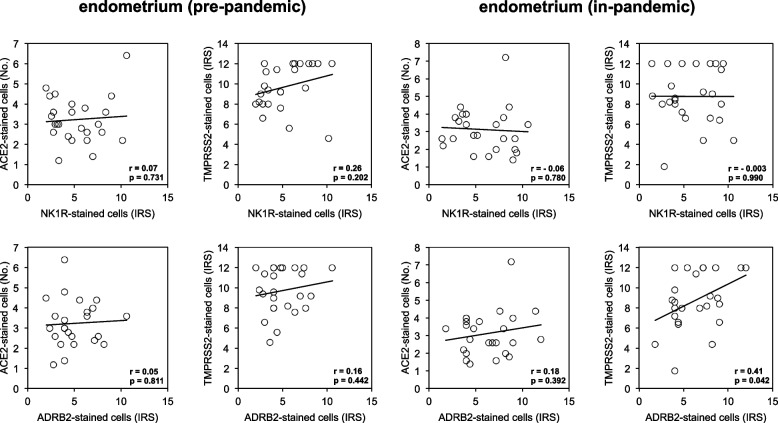
Shows correlation between ACE2 or TMPRSS2 and NK1R or ADRB2 expressions in endometrial samples that were retrieved in pre-pandemic period (left panel) and in in-pandemic period (right panel). There was no significant correlation between any of these markers in these two groups of endometria except ADRB2 and TMPRSS2 expression. A significant correlation was observed in the expression between ADRB2 and TMPRSS2 (*r* = 0.41, *p* = 0.042) in the in-pandemic endometria

We were interested to know whether a variable degree of anxiety/stress during Covid-19 pandemic might increase the expression pattern of ACE2 and TMPRSS2 comparing to those in endometria collected before pandemic. Pearson product-moment correlation coefficient analysis showed a lack of correlation in the expression between NK1R or ADRB2 and ACE2 or TMPRSS2 in pre-pandemic endometria (Fig. 3, left panel). In contrast, a significant correlation in the expression between ADRB2 and TMPRSS2 (r-0.41, *p* = 0.042) was found in in-pandemic endometria (Fig. 3, right panel). However, this correlation was lost between ACE2 and NK1R, between ACE2 and ADRB2, or between NK1R and TMPRSS2 expression in endometria that were collected during the period of pandemic (Fig. 3, right panel).

### Distribution of CD68- and MPO-immunoreactive cells in endometria

As a measure of acute or chronic tissue inflammatory reaction, we analyzed tissue accumulation of CD68- and MPO-stained cells in endometria by immunohistochemistry collected from women in 2019 (pre-pandemic) and 2020 (in-pandemic) in order to examine any change in endometrial inflammation. An image of CD68- and MPO-stained slides and corresponding positive controls (lymph node for each of CD68 and MPO) and negative controls is shown in Suppl. Figure 1A. Both CD68-stained macrophages and MPO-stained neutrophils were found in the stromal compartment of endometria. Mann–Whitney U test indicated that there were no significant differences in the number of CD68-stained cells and MPO-stained cells in the endometria between pre-pandemic group and in-pandemic group (Suppl. Figure 1B and Suppl. Figure 1C).

### Correlation between CD68 or MPO and NK1R or ADRB2 expressions in endometria

To investigate the fact that pandemic-related environmental stress may induce a variable amount of tissue inflammatory reaction in endometria as a protective measure and this could be a source of tissue stress reaction, we analyzed a possible correlation between CD68 or MPO and NK1R or ADRB2 expressions in the endometria of pre-pandemic and in-pandemic group. No correlations were observed in the expressions between CD68 and ADRB2 or NK1R or between MPO and ADRB2 or NK1R in endometria that were collected in pre-pandemic or in in-pandemic period (Suppl. Figure 2).

Analysis of covariance (ANCOVA) with 7 confounding variables (age, BMI, gravidity, parity, smoking, hormonal therapy, and menstrual cycle) as covariates was used to compare expression pattern of each marker (ACE2, TMPRSS2, NK1R, ADRB2, CD68, MPO) in endometria that were collected from cases before (pre-pandemic) and during Covid-19 pandemic (in-pandemic). Among these six markers, a significant difference was found between the two groups in ADRB2 expression (*P* = 0.015) after adjusting for covariates (Table 4) indicating possible occurrence of a tissue stress reaction in the endometria during the Covid-19 pandemic.

**Table 4:** Immunostaining levels of ACE2, TMPRSS2, NK1R, ADRB2, CD68, and MPO in endometria collected from cases before and during Covid-19 pandemic

	Endometrium (pre-pandemic) (*n* = 25)	Endometrium (in-pandemic) (*n* = 25)	*P* value
**ACE2**-stained cells (mean ± SEM)	3.2 ± 0.2	3.1 ± 0.3	0.718
Median	3.0	2.8	
Range of IRS	1.2–6.4	1.4–7.2	
**TMPRSS2**-stained cells (mean ± SEM)	9.7 ± 0.4	8.8 ± 0.6	0.519
Median	9.6	8.6	
Range of IRS	4.6–12.0	1.8–12.0	
**NK1R** (IRS) (mean ± SEM)	5.3 ± 0.5	5.9 ± 0.6	0.283
Median	4.8	5.2	
Range of IRS	2.0–10.6	1.4–10.6	
**ADRB2** (IRS) (mean ± SEM)	5.0 ± 0.4	6.3 ± 0.5	**0.015**
Median	4.0	6.4	
Range of IRS	2.0–10.6	1.8–12.0	
**CD68**-stained cells (mean ± SEM)	84.2 ± 9.1	99.6 ± 13.5	0.779
Median	79.2	90.6	
Range of IR cells	9.8–179.2	0–287.6	
**MPO**-stained cells (mean ± SEM)	22.3 ± 6.8	41.1 ± 9.2	0.202
Median	10.6	16.2	
Range of IR cells	1.6–169	0–179	

*ACE2* angiotensin-converting enzyme 2, *TMPRSS2* trans-membrane serine protease 2, *NK1R* neurokinin receptor 1, *ADRB2* beta 2-adrenergic receptor, *CD68* marker of macrophages, *MPO* myeloperoxidase, marker of neutrophils, *IR* immunoreactive cells, *IRS* immunoreactive score

^*^P value after adjusting for covariates by ANCOVA (analysis of covariance)

### Age-dependent distribution of different markers in endometria

**Fig. 4:**
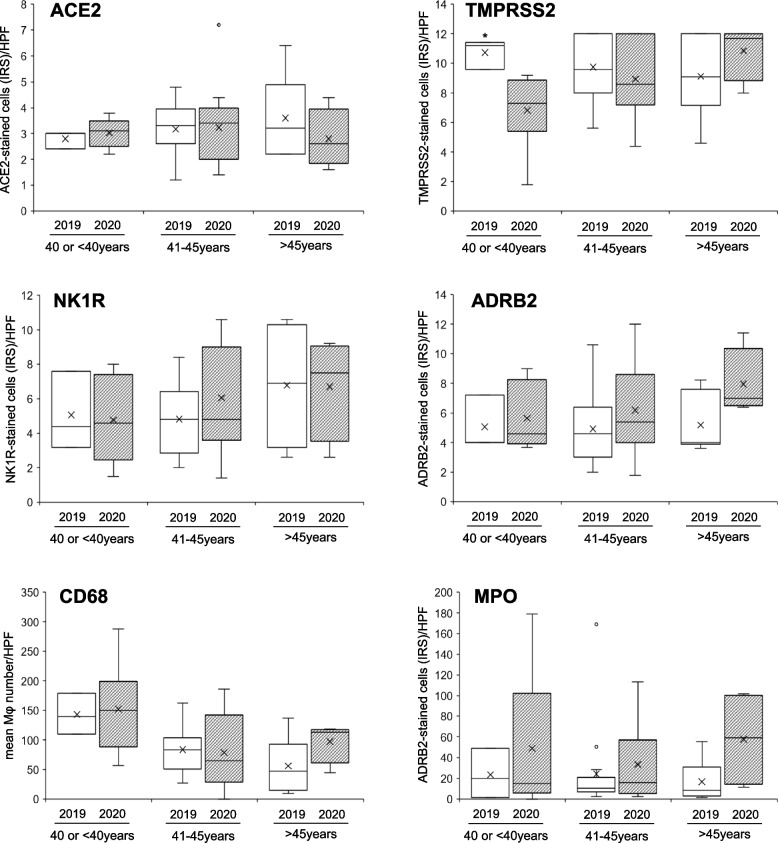
Shows age-dependent distribution in the expression profiles of ACE2 and TMPRSS2, NK1R and ADRB2, and CD68 and MPO in the endometria that were collected before pandemic (2019) and during pandemic (2020) period. Among all these investigated markers, a statistically significant difference was found only for TMPRSS expression (*P = 0.028) in endometria of pre-pandemic (2019) women in their ≤ 40 years of age comparing to that in in-pandemic (2020) endometria of women in the similar age groups. An increasing trend in the expression of TMPRSS2 was observed in in-pandemic endometria (2020) than that in pre-pandemic endometria (2019) among cases with higher ages (> 45 years). The boxes represent the interquartile ranges, horizontal lines in the boxes represent median values, and cross (x) marks in the boxes indicates mean values

With the speculation in mind that expression profiles of ACE2, TMPRSS2, NK1R, ADRB2, and CD68, MPO in endometria, that were collected during pre- and in-pandemic period, may differ in different age groups, we evaluated the immunoreactivity against all these markers in the endometria among three age groups, ≤ 40 years, 41–45 years and > 45 years (Fig. 4). Among all these investigated markers, a statistically significant difference was found only for TMPRSS2 expression (*P* = 0.028) in the endometria of pre-pandemic (2019) group in their ≤ 40 years of age comparing to that in in-pandemic (2020) group in the similar age groups. The expression of TMPRSS2 showed an increasing tendency during the period of pandemic than that in pre-pandemic endometria among the higher age group (> 45 years) (IRS: median 11.7 vs. 9.1) (Fig. 4, right upper panel).

Kruskal–Wallis test among three different age groups indicated that there was no significant difference in the expression of any of these six markers in two groups of endometria except CD68 expression. The tissue infiltration of CD68-stained cells in the endometria of pre-pandemic group was significantly decreased in higher age group (> 45 years) (median, 47.1/HPF) (*P* = 0.024) comparing to that of women in other two age groups [≤ 40 years (median, 139.8/HPF) and 41–45 years (median, 83.4/HPF)] (Suppl. Table 2).

## Discussion

The decreasing trend in the infection and, more importantly, much reduced mortality rate of SARS-CoV-2 infection in the majority of countries have generated great relief in mental, psychological and physical stress. However, we should not forget the global spread of SARS-CoV-2 infection with the onset of 2020 with serious effect on mental and physical health among general population including women in their reproductive age. In this retrospective cohort study, we investigated, for the first time, the association between tissue stress reaction in response to mental and/or physical stress/anxiety and SARS-CoV-2 cell entry proteins in endometria of women that were collected before and during the Covid-19 pandemic. Our serial experiments with immunohistochemical analysis revealed that there were no significant differences in ACE2 and TMPRSS2 expressions and revealed a lack of co-expression between these two cell entry proteins in the endometria between these two groups of women. The similar expression profiles of ACE2 and TMPRSS2 in endometria before and during the pandemic were found irrespective of phases of the menstrual cycle and age of the women.

With the speculation in mind that a state of tissue stress reaction in response to fear/anxiety/worry in women during this pandemic may affect the results, we found that all these women during the two time periods (2019 and 2020) suffer from a variable tissue stress reaction in their endometria as manifested by the variable tissue expression of adrenoreceptor ADRB2 and NK1R. Interestingly, the endometrial expression of ADRB2 was significantly higher during the pandemic period as compared to that before the pandemic with a positive correlation between ADRB2 and TMPRSS2 expression in the endometria of in-pandemic group. The significantly higher expression of ADRB2 in in-pandemic endometria persisted even after adjusting for different covariates by ANCOVA. These findings indicate that TMPRSS2 expression in endometria that were collected during the pandemic period may be affected by ADRB2 expression. However, a cause-effect relationship in this finding is unclear and needs future study for further clarification and strengthen our current findings.

We can propose some possible mechanisms that may clarify the induction of SARS-CoV-2 viral entry proteins: (i) direct or indirect effect of tissue stress reaction in the endometria in response to exogenous mental anxiety and stress due to the pandemic, (ii) although not applicable for this study, SARS-CoV-2 infection-provoked inflammatory process, such as type-1 interferon 1 (IFN-1) production and formation of neutrophil extracellular traps (NET) among others, could be another factor to up-regulate ACE2 and/or TMPRSS2 expression [28, 29], (iii) Stress-mediated signaling pathway may activate IFN-1 resulting in the induction of SARS-CoV-2 viral entry proteins, a cascade very similar to SARS-CoV-2 infection. In fact, a recent study demonstrated that cellular stress signaling activates and secretes IFN-1 in response to elevated oxidative stress [30]. Future study may clarify this issue.

As a first-line defensive cells that might be emerged in and protective for endogenous tissues during this pandemic, we did not find any difference in the accumulation of two innate immune cells, i.e. macrophages and neutrophils, in the endometria collected from women during the pre-pandemic (2019) and in-pandemic period (2020). In fact, any emergency crisis in external or internal environment of human body may increase the tissue infiltration of innate immune cells in internal organs including the uterus [31, 32]. In addition, no correlations were observed in the expression profiles between CD68 and ADRB2 or NK1R or between MPO and ADRB2 or NK1R in endometria that were collected in these two time periods. To our knowledge, no previous study demonstrated a relationship between tissue inflammatory reaction and tissue-stress reaction in human endometria.

The age-dependent significantly decreased number of CD68-stained cells in pre-pandemic endometria may be the natural consequence of declined innate immunity among the higher age groups. From these findings we can presume that although pandemic-related environmental stress may induce a variable amount of inflammatory reaction in endometria, this was not the cause of additional tissue stress reaction.

While the increase in endometrial staining of ADRB2 in women of in-pandemic group as compared with the pre-pandemic group does not seem to impact other markers that we evaluated, it is possible it may contribute to menstrual irregularity [21, 22], decreased duration of menstruation and volume of menstrual blood loss [23], and newly onset of dysmenorrhea [22]. Indeed, it has been reported that anxiety is a determinant of menstrual irregularity [33, 34].

It is unclear what effect, if any, the Covid-19 pandemic will have on male and female fertility and reproductive function. Until now, several lines of evidence using single-cell sequencing datasets demonstrated conflicting results on the expression of ACE2 and TMPRSS2 in different organs such as lung, cornea, ileum, colon, heart, gallbladder including uterus, ovary, breast, and testis [15, 16]. A recent report with transcriptomic and proteomic analyses failed to detect expression and co-expression of ACE2 and TMPRSS2 in human endometria [15]. However, any report on the expression of known viral host entry proteins in endometria collected from women of two different time frames of pandemic is unknown. Most of the studies reported a lack of co-expression between ACE2 and TMPRSS2 in different reproductive tissues that interrupts efficient SARS-CoV-2 entry into target cells. Our current finding is a further piece of biological evidence that lack of correlation persists in the expression ACE2 and TMPRSS2, at the protein levels, in the endometria of women that were collected during the height of the Covid-19 pandemic.

Although a variable degree of exogenous anxiety/stress for pandemic may strengthen the correlation in the expression between ADRB2 and TMPRSS2 in endometria, this correlation was not found in pre-pandemic endometria. These findings may suggest that suffering from any environmental anxiety/stress (the effect of substance P or catecholamine) may make it easy for SARS-CoV-2 to enter the cell by increasing the expression of cell entry proteins. On the other hand, an increase in the expression of SARS-CoV-2 cell entry proteins may induce the expression of NK1R and ADRB2 by an unknown etiology. Despite this correlation in the expression between anxiety/stress receptor and SARS-CoV-2 cell entry receptor such as TMPRSS2, we did not find any correlation between expression of ACE2 and TMPRSS2 in the endometria of these two groups of women. In fact, co-expression of ACE2 with TMPRSS2 or with other proteases is necessary to facilitate the entry of SARS-CoV-2 into the host cells. Our findings might be reassuring to the women in their reproductive age who experience a variable degree of stress/anxiety and are planning to conceive either naturally or by artificial reproductive technology (ART) during this pandemic. In addition, an increased tissue stress reaction in in-pandemic endometria may be associated with different menstruation-related disturbances in women who suffer from variable psychological anxiety and stress during the Covid-19 pandemic. Further studies are warranted to precisely clarify these unclear issues on the Covid-19 pandemic-related environmental stress/anxiety, tissue stress reaction in the endometria, expression profiles of SARS-CoV-2 viral entry proteins and their consequence on the reproductive function and/or reproductive outcome.

One question still remains to be addressed. What is the biological significance of similar neurogenic receptor (NK1R) expression and dissimilar adrenergic receptor (ADRB2) expression in the endometria between pre- and in-pandemic period? Conditional and unconditional reflex of fear/anxiety/stress may result in stressful insult to our body including female reproductive organs. For example, surgery for any gynecological and non-gynecological indication inevitably results in tissue damage, trauma, or stress to the body as mediated by adrenergic and neurogenic pathway [35]. In response to tissue stress reaction either by surgery or fear/anxiety-mediated stress during Covid-19 pandemic, various bioactive molecules such as catecholamines are secreted that are known to suppress cell-mediated immunity and promote angiogenesis and metastasis in animal models [17, 18, 36, 37]. A recent interesting study demonstrated that tissue stress reaction induced by surgery activates adrenergic signaling, increases angiogenesis and accelerates the growth of endometriosis in the mouse model [35]. Similarly a state of fear/anxiety/stress among reproductive women during Covid-19 pandemic may trigger the release of catecholamines from adrenal glands/sensory/sympathetic nerves and neuropeptide such as substance-P from sensory nerves [19, 36]. Our current findings may at least indicate that possible release of neurotransmitters/neuropeptide (catecholamines/substance-P) and their interaction with respective receptors in endometria may promote the growth and progression of any coexistent disease, such as endometriosis, in women who suffer from persistent stress. Although we did not evaluate stress-related hormone levels per se in these two groups of women as a measure of stress hormone response, the increased stress has been documented in women during the pandemic [23] and increased levels of adrenaline and noradrenaline was observed in serum after pelvic surgery [38]. It is unclear whether endometrial expressions of ADRB2 and NK1R are involved in the worsening of coexisting diseases. In fact, a proportion of our study population had coexistent uterine fibroids, endometriosis and adenomyosis. Future study may clarify the cause-effect relationship between pandemic related stress hormones and progression of reproductive diseases.

A controversial issue needs to be resolved in our current study. Based on the information of several recent studies [35, 39, 40], how can we avoid the bias of surgery-related induction of ADRB2 expression in the in-pandemic endometria in our current study? As a matter of fact, all these surgery-induced stress related events with increased ADRB2 expression occurred under the manipulation of the pathological lesions. In our current study, all tissues were collected from the hysterectomy specimen without any surgical manipulation of the endometrium. Although the procedure of hysterectomy may elicit a substantial amount of stress reaction in the uterus such as in myometrium and/or in endometrium, we presume that all collected endometrial tissues in this study were under minimal stress of surgery. Considering this assumption, we believe that we can at least exclude the major bias of surgery-related stress reaction in the pre-pandemic and in-pandemic endometrium. Our knowledge on this issue is still insufficient. In order to address this issue, further study is needed to confirm the difference between surgery (hysterectomy)-related and exogenous pandemic-related tissue stress reaction in the endometrium.

It has been reported that cathepsin L (CTSL) is expressed by several cancerous tissues that regulates cancer progression and increases the susceptibility to SARS-CoV-2 infection [41–43]. In addition, colorectal cancer and lung cancer tissues highly express ACE2 and TMPRSS2 resulting in severe symptoms and/or unfavorable prognosis of SARS-CoV-2 infection [44, 45]. On the other hand, a recent study analyzed SARS-CoV-2 infection-related gene expression (*ACE2, TMPRSS2, CTSB, CTSL*) from endometrial transcriptomic data sets [46]. Based on differential expression of these genes, the authors suggested that endometrial tissue is likely safe from SARS-CoV-2 cell entry. The lack of a correlation between ACE2 and TMPRSS2 protein expression in the in-pandemic endometrium in our current study coincided with this report. When we consider the risk–benefit in the expression profiles of SARS-CoV-2 entry proteins, we believe that groups of women, with expression of ACE2 and TMPRSS2 proteins in their endometria during the period of pandemic, might be safe as well even a small proportion of these women contain some malignant tumors. In our separate analysis, we did not find any significant difference in the expression profiles of ACE2 and TMPRSS2 in the endometria of women with and without malignant tumors (data not shown).

There are some biological and clinical significance of our current findings. (1) We can assure and reassure the women suffering from fear/anxiety/stress during current Covid-19 pandemic that they can be safe to make a plan who desire to conceive naturally or by ART. (2) Regarding fear/anxiety of successful implantation and placentation during current pandemic, our findings of a lack of co-expression between ACE2 and TMPRSS2 in endometria may elute this anxiety among reproductive women. These findings may also be useful to ART practitioners to make a safe decision. (3) Our findings of stress-related receptors expression (ADRB2 and NK1R) in endometria may indicate the importance of proper counseling with women who harbor any coexistent disease in their uterus or who suffer from any menstruation-related problem.

The main strength of this study is that we retrieved tissue blocks and analyzed target markers in endometria during two different time frames, pre-pandemic (2019) and during the pandemic (2020). In addition, we demonstrated for the first time the association between the expression of stress-reactive proteins and expression of two SARS-CoV-2 viral entry proteins in the endometria collected during two different times. This is in contrast to a previous study that analyzed differential expression of virus infection-related genes (*ACE2, TMPRSS2, CTSB, CTSL*) from endometrial transcriptomic data sets [46] but did not compare their protein expression profiles in endometrial tissue samples collected during pre-pandemic and in-pandemic period.

There are some limitations in this study: (1) This is a retrospective observational cohort study with small sample size and our analysis was confined to immunohistochemistry; (2) Our results cannot rule out the possibility that proteases other than TMPRSS2 including cysteine protease cathepsin L (CatL, encoded by the gene *CTSL*) may facilitate viral entry in some endometrial cells; (3) The study population described are women in their late menstrual period, who are generally prone to suffer from menstrual irregularities. Thus, our findings may not be over generalized to women of childbearing age (15–35 years). Further study may clarify this issue; (4) We could not measure serum levels of stress-related hormones between these two groups of women nor did we evaluate stress/anxiety scores by questionnaire survey. However, our hypothesis is corroborated with one recent study demonstrating that Covid-19 pandemic positively influenced both mental anxiety and stress levels [47]. (5) Due to retrospective nature of this study, we could not perform either antigen test or PCR test to identify SARS-CoV-2 viral infection in these two groups of women. Based on the differential expression of virus infection-related genes (*ACE2, TMPRSS2, CTSB, CTSL*), a recent study claimed that endometrial tissue is likely safe from SARS-CoV-2 cell entry but susceptibility increases with age [46]. This finding coincides with our current study that showed an increasing expression tendency of TMPRSS2 in advancing age group than that in younger age group during the pandemic period (Fig. 4). This may partly explain why individuals of older age are more susceptible to SARS-CoV-2 infection. Further study with collection of large sample size may address these unclear issues.

## Conclusions and recommendations

We demonstrated expression profiles of two SARS-CoV-2 cell entry proteins at the protein level and a lack of co-expression between ACE2 and TMPRSS2 in endometria collected from women before and during Covid-19 pandemic. These findings may be reassuring to women in their reproductive age that they are not more susceptible to infection by SARS-CoV-2 and suggest that stressful women during this pandemic can safely decide to conceive naturally or by ART. In clinical practice, a non-painful procedure of endometrial tissue collection by pipelle method may help clinicians to analyze ACE2 and TMPRSS2 by immunohistochemistry. The endometrial sampling using pipelle is quick, safe, accurate and cost-effective outpatient procedure, which avoids general anesthesia and has a high sensitivity and specificity for the detection of endometrial hyperplasia and malignancy [48].

The Covid-19 pandemic created a significant impact on medically assisted reproduction (MAR). During conduction of this study, governments around the world announced far-reaching restrictions in personal freedom and medical services due to Covid-19 [49]. With a solid consensus, the key recommendations for practitioners include suspension of ovulation induction, intrauterine insemination, in vitro fertilization as well as non-urgent gamete cryopreservation, cancellation of all embryo transfers, whether fresh or frozen, and suspension of elective surgery and non-urgent diagnostic procedures [50, 51]. Another concern is that a prolonged lockdown of fertility treatment can be detrimental to patients and society. This is applicable for a subgroup of infertile women, in particular, patients with low prognosis for success in ART who tends to lose their fertility potential rapidly [49]. The ESHRE Covid-19 working group suggested individualized management and adaptation of ART services and treatment planning [52]. A recent report proposed that personalizing medication for ovarian stimulation and elective freezing of oocytes or embryos should be the first choice during the current emergency period [49]. Our current findings may provide some additional piece of evidence to understand the safety of ART intervention and outcome during the pandemic and to recommend medical practitioners to undertake individualized treatment plan for women who wish for baby during current pandemic.

Proper counseling to women in their reproductive age is necessary to alleviate unnecessary anxiety/stress during this pandemic in order to resolve their menstrual problems, if any. Given the fact that environmental stress/anxiety increases angiogenesis and accelerates the progression of coexistent diseases, our findings of a variable amount of tissue stress reaction in the endometria may recommend the importance of proper counseling with women who harbor any coexistent disease in their uterus during Covid-19 pandemic. Future prospective studies with large sample size are warranted to address these issues and to strengthen our current findings.

## Supplementary Information

**Additional file 1. Suppl. Table 1.** Pathological diagnosis of cases from whom endometrial samples were collected before and during Covid-19 pandemic

**Additional file 2. Suppl. Table 2.** Age-dependent distribution of markers expressed in endometria that were collected during pre-pandemicand in-pandemic period.

**Additional file 3. Suppl. Fig. 1.** An image of slides showing the hematoxyline eosin-stained and immunohistochemical analysis of macrophage marker, CD68- and neutrophils marker, myeloperoxidase-stained cells in the pre-pandemicand in-pandemic endometriawith corresponding positive controlsand negative controls.Shows the number of CD68- and MPO-stained cells in the stromal compartment per high power fieldin pre-pandemic and in-pandemic endometria. Mann-Whitney U test indicated no significance difference in the tissue infiltration of CD68-stained macrophages and MPO-stained neutrophils between these two groups of endometria. The boxes represent the interquartile ranges and horizontal lines in the boxes represent median values. Scale bar = 50μm for each slide.

**Additional file 4. Suppl. Fig. 2.** Shows correlation between CD68 or MPO-stained cells and NK1R or ADRB2 expressions in endometrial samples that were retrieved during pre-pandemic periodand in-pandemic period. There was no significant correlation between any of these markers in either pre-pandemic endometria or in in-pandemic endometria.

## Data Availability

The datasets generated and/or analyzed during the current study are not publicly available due to ethical concerns but are available from the corresponding author on reasonable request.

## Abbreviations

Covid-19: Coronavirus disease 19
SARS-CoV-2: Severe acute respiratory syndrome coronavirus 2
ACE2: Angiotensin-converting enzyme 2
TMPRSS2: Transmembrane serine protease 2
ADRB2: Adrenergic receptor β2
NK1R: Neurokinin 1 receptor
Mφ: Macrophages
MPO: Myeloperoxidase
IRS: Immune-reactive score
CatL: Cysteine protease cathepsin L
CatB: Cysteine protease cathepsin B
IFN-1: Type-1 interferon
NET: Neutrophil extracellular traps
ART: Artificial reproductive technology
