# Supplementary material for: Association between tissue stress reaction and ACE2/TMPRSS2 expression in endometria of reproductive aged women before and during Covid-19 pandemic
Source: BMC Womens Health. 2023 May 4;23:229. doi: 10.1186/s12905-023-02378-0 (PMC10158702; doi:10.1186/s12905-023-02378-0)
Supplement: Supplementary file 1 — Additional file 1. Suppl. Table 1. Pathological diagnosis of cases from whom endometrial samples were collected before and during Covid-19 pandemic [file 12905_2023_2378_MOESM1_ESM.docx]

Suppl. Table 1. Pathological diagnosis of cases from whom endometrial samples were collected before and during Covid-19 pandemic

| Case number Pre-pandemic In-pandemic  (n=25) (n=25) |
| --- |
| 1. HSIL, CIN3, pTis15*12mm leiomyoma, endometriosis   leiomyoma ectopic endometrium in cervix  2. HSIL, CIN3, leiomyoma leiomyoma  3. HSIL, CIN3, leiomyoma, HSIL, CIN3, leiomyoma,  adenomatoid tumor (rt. fimbriae) adenomyosis, endometrial polyp  4. CIN3 HSIL, CIN2, leiomyoma, adeno  5. no residual tumor, SCC IA1 HSIL, CIN3, leiomyoma, adeno,  adenomatoid tumor (rt.corpus)  6. leiomyoma leiomyoma  7. leiomyoma, adenomyosis leiomyoma  8. leiomyoma no residual tumor  9. AIS, pTis37*11mm, CIN2-3, leiomyoma  leiomyoma  10. leiomyoma no neoplasm, endometriosis  11. adenomyosis leiomyoma  12. multiple leiomyomas, paratubal leiomyoma  cyst (lt. Fallopian tube)  13. leiomyoma adenomyosis  14. leiomyoma leiomyoma, endometriosis  15. leiomyoma, adenomyosis adenomyosis  16. leiomyoma, adenomyosis leiomyoma  17. HSIL, CIN3, leiomyoma HSIL, CIN3, endometrial polyp  18. leiomyoma, endometriosis leiomyoma, endometriosis  19. leiomyoma, endometriosis endometriosis, endometrial polyp  20. CIN1, leiomyoma leiomyoma  21. leiomyoma, endometrial polyp leiomyoma, adenomyosis  22. leiomyoma leiomyoma  23. leiomyoma adenomyosis  24. leiomyoma leiomyoma  25. leiomyoma leiomyoma |

HSIL, high grade squamous intraepithelial lesion; CIN1-3, cervical intraepithelial neoplasia, grade 1-3; SCC, squamous cell carcinoma; AIS, cervical adenocarcinoma in situ; adeno, adenomyosis.
