# Supplementary material for: Association between tissue stress reaction and ACE2/TMPRSS2 expression in endometria of reproductive aged women before and during Covid-19 pandemic
Source: BMC Womens Health. 2023 May 4;23:229. doi: 10.1186/s12905-023-02378-0 (PMC10158702; doi:10.1186/s12905-023-02378-0)
Supplement: Supplementary file 2 — Additional file 2. Suppl. Table 2. Age-dependent distribution of markers expressed in endometria that were collected during pre-pandemicand in-pandemic period. [file 12905_2023_2378_MOESM2_ESM.docx]

Suppl. Table 2. Age-dependent distribution of markers expressed in endometria that were collected during pre-pandemic (2019) and in-pandemic period (2020).

|  | Age | | | p value* |
| --- | --- | --- | --- | --- |
|  | 40 or <40years | 41-45years | >45years |  |
| **Pre-pandemic (2019)** | n=3 | n=16 | n=6 |  |
| **ACE2** (mean ± SEM) | 2.8 ± 0.2 | 3.2 ± 0.3 | 3.6 ± 0.7 | 0.792 |
| Median (range) | 3.0 (2.4-3.0) | 3.3 (1.2-4.8) | 3.2 (2.2-6.4) |  |
| **TMPRSS2** (mean ± SEM) | 10.7 ± 0.6 | 9.7 ± 0.6 | 9.1 ± 1.1 | 0.805 |
| Median (range) | 11.2 (9.6-11.4) | 9.6 (5.6-12) | 9.1 (4.6-12) |  |
| **NK1R** (mean ± SEM) | 5.1 ± 1.3 | 4.8 ± 0.5 | 6.8 ± 1.5 | 0.446 |
| Median (range) | 4.4 (3.2-7.6) | 4.8 (2.0-8.4) | 6.9 (2.6-10.6) |  |
| **ADRB2** (mean ± SEM) | 5.1 ± 1.1 | 4.9 ± 0.6 | 5.2 ± 0.8 | 0.943 |
| Median (range) | 4.0 (4.0-7.2) | 4.6 (2.0-10.6) | 4.0 (3.6-8.2) |  |
| **CD68** (mean ± SEM) | 142.9 ± 20.2 | 83.8 ± 9.4 | 55.9 ± 19.1 | **0.024** |
| Median (range) | 139.8 (109.6-179.2) | 83.4 (26.8-162.2) | 47.1 (9.8-136.6) |  |
| **MPO** (mean ± SEM) | 23.5 ± 13.8 | 24.1 ± 10.1 | 16.7 ± 8.5 | 0.830 |
| Median (range) | 20.0 (1.6-49) | 10.6 (2.4-169) | 8.3 (1.6-55.8) |  |
|  |  |  |  |  |
| **In-pandemic (2020)** | n=6 | n=15 | n=4 |  |
| **ACE2** (mean ± SEM) | 3.0 ± 0.2 | 3.2 ± 0.4 | 2.8 ± 0.6 | 0.861 |
| Median (range) | 3.1 (2.2-3.8) | 3.4 (1.4-7.2) | 2.6 (1.6-4.4) |  |
| **TMPRSS2** (mean ± SEM) | 6.8 ± 1.1 | 9.0 ± 0.7 | 10.9 ± 1.0 | 0.130 |
| Median (range) | 7.3 (1.8-9.2) | 8.6 (4.4-12) | 11.7 (8.0-12) |  |
| **NK1R** (mean ± SEM) | 4.8 ± 1.0 | 6.1 ± 0.7 | 6.7 ± 1.5 | 0.532 |
| Median (range) | 4.6 (1.5-8.0) | 4.8 (1.4-10.6) | 7.5 (2.6-9.2) |  |
| **ADRB2** (mean ± SEM) | 5.7 ± 0.9 | 6.2 ± 0.7 | 8.0 ± 1.2 | 0.523 |
| Median (range) | 4.6 (3.7-9.0) | 5.4 (1.8-12) | 7.0 (6.4-11.4) |  |
| **CD68** (mean ± SEM) | 152.4 ± 31.9 | 79.1 ± 15.8 | 97.4 ± 17.5 | 0.121 |
| Median (range) | 150.2 (57.2-287.6) | 64.4 (0.0-186) | 113.3 (45-118) |  |
| **MPO** (mean ± SEM) | 49.0 ± 28.3 | 33.5 ± 9.2 | 57.9 ± 23.7 | 0.495 |
| Median (range) | 14.9 (0.0-179) | 16.2 (2.2-113.6) | 59.1 (11.4-102) |  |

* Data were analyzed by Kruskal-Wallis test.
