# Supplementary figures and images for: Association between tissue stress reaction and ACE2/TMPRSS2 expression in endometria of reproductive aged women before and during Covid-19 pandemic
Source: BMC Womens Health. 2023 May 4;23:229. doi: 10.1186/s12905-023-02378-0 (PMC10158702; doi:10.1186/s12905-023-02378-0)

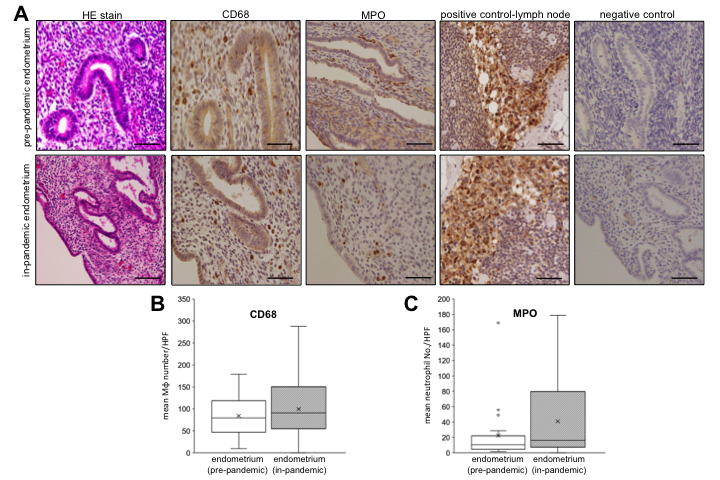

Supplement: Supplementary file 3 — Additional file 3. Suppl. Fig. 1. An image of slides showing the hematoxyline eosin-stained and immunohistochemical analysis of macrophage marker, CD68- and neutrophils marker, myeloperoxidase-stained cells in the pre-pandemicand in-pandemic endometriawith corresponding positive controlsand negative controls.Shows the number of CD68- and MPO-stained cells in the stromal compartment per high power fieldin pre-pandemic and in-pandemic endometria. Mann-Whitney U test indicated no significance difference in the tissue infiltration of CD68-stained macrophages and MPO-stained neutrophils between these two groups of endometria. The boxes represent the interquartile ranges and horizontal lines in the boxes represent median values. Scale bar = 50μm for each slide. [file 12905_2023_2378_MOESM3_ESM.tiff]

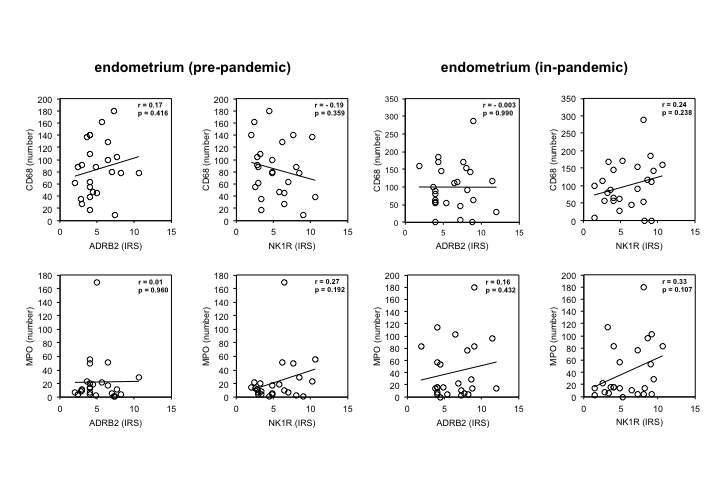

Supplement: Supplementary file 4 — Additional file 4. Suppl. Fig. 2. Shows correlation between CD68 or MPO-stained cells and NK1R or ADRB2 expressions in endometrial samples that were retrieved during pre-pandemic periodand in-pandemic period. There was no significant correlation between any of these markers in either pre-pandemic endometria or in in-pandemic endometria. [file 12905_2023_2378_MOESM4_ESM.tiff]
